# Bisecting N-Acetylglucosamine Structures Inhibit Hypoxia-Induced Epithelial-Mesenchymal Transition in Breast Cancer Cells

**DOI:** 10.3389/fphys.2018.00210

**Published:** 2018-03-09

**Authors:** Zengqi Tan, Chenxing Wang, Xiang Li, Feng Guan

**Affiliations:** ^1^College of Life Science, Northwest University, Xi'an, China; ^2^School of Biotechnology, Jiangnan University, Wuxi, China; ^3^Wuxi Medical School, Jiangnan University, Wuxi, China

**Keywords:** hypoxia, EMT, MGAT3, bisecting GlcNAc structures, breast cancer

## Abstract

The epithelial-mesenchymal transition (EMT) process plays a key role in many biological processes, including tissue fibrosis, metastatic diseases, and cancer progression. EMT can be induced by certain factors, notably hypoxia, in the tumor microenvironment. Aberrant levels of certain N-glycans is associated with cancer progression. We used an integrated strategy (mass spectrometry in combination with lectin microarray analysis) to elucidate aberrant glycosylation in a hypoxia-induced EMT model using breast cancer cell lines MCF7 and MDA-MB-231. The model showed reduced levels of bisecting GlcNAc structures, and downregulated expression of the corresponding glycosyltransferase MGAT3. MGAT3 overexpression in MCF7 suppressed cell migration, proliferation, colony formation, expression of EMT markers, and AKT signaling pathway, whereas MGAT3 knockdown (shRNA silencing) had opposite effects. Our findings clearly demonstrate the functional role (and effects of dysregulation) of bisecting GlcNAc structures in hypoxia-induced EMT, and provide a useful basis for further detailed studies of physiological functions of these structures in breast cancer.

## Introduction

Breast cancer comprises ~30% of new cancer diagnoses, and is one of the most common causes of cancer mortality in women (Siegel et al., 2017). Many (25–40% of) invasive breast cancers contain hypoxic regions (Lundgren et al., 2007). Hypoxia (low oxygen level) is a well-documented characteristic of the tumor microenvironment. In patients with various types of cancer, tumors under hypoxic conditions were associated with increased metastasis and poor survival (Höckel and Vaupel, 2001). Most cellular responses to hypoxia are regulated by hypoxia-inducible factor-1α (HIF-1α) (Keith and Simon, 2007). HIF-1 is a heterodimer, which is consist of hypoxic response factor HIF-1α and the constitutively expressed HIF-1β. In normoxia, HIF-1α is modified by prolyl hydroxylase, and hydroxylated HIF1α is bound to the Von Hippel-Lindau (VHL) complex. VHL mediates the ubiquitylation of HIF-1α, and contribute to degradation of HIF-1α. In hypoxia, HIF-1 remains stable, and stabilized HIF-1α is translocated to the nucleus, where it binds to hypoxia-response elements (HREs) and activates expression of hypoxia-response genes that control cellular processes such as energy metabolism, neovascularization, survival, pH, and migration (Pouysségur et al., 2006).

Hypoxia has also been shown to induce the epithelial-mesenchymal transition (EMT) process (Liu L. et al., 2014; Liu Y. et al., 2014). Epithelial cells that undergo EMT are characterized by loss of cell-cell adhesion and cell polarity, and acquisition of migratory and invasive properties, and become involved in wound healing, tissue regeneration, organ fibrosis and tumor progression (Marcucci et al., 2016). Numerous studies have demonstrated functional roles and altered levels of glycans and their glycogenes in EMT and various other physiological and pathological processes. Reduced levels of β4GalT4 gene and its product gangliotetraosylceramide (Gg4) were observed during transforming growth factor (TGFβ)-induced EMT in normal mouse mammary epithelial NMuMG cells (Guan et al., 2009). Expression of N-acetylglucosaminyltransferase III (GnT-III, also known as MGAT3) and its products (bisecting GlcNAc structures) were suppressed in TGFβ-induced EMT (Xu et al., 2012; Tan et al., 2014). Bisecting GlcNAc structures were present in membrane proteins of serous ovarian cancer cells but not of non-cancerous ovarian surface epithelial cells (Anugraham et al., 2014). MGAT3 expression was upregulated in brains of Alzheimer's disease patients (Akasaka-Manya et al., 2010).

Bisecting GlcNAc structure, a modification found in complex and hybrid N-glycans, results from attachment of N-acetylglucosamine (GlcNAc) to core mannose of the N-glycan via β1,4 inkage, catalyzed by MGAT3. Bisecting GlcNAc structures play regulatory roles in biosynthesis of N-glycans, and their presence suppresses continued processing or elongation of N-glycans catalyzed by other glycosyltransferases (e.g., GnT-II, GnT-IV, GnT-V) (Xu et al., 2011, 2012; Lu et al., 2016). GnT-V and its products (β1,6-branched N-glycans) were positively correlated with tumor cell metastasis, whereas MGAT3, as an antagonist of GnT-V, suppressed metastasis (Gu and Taniguchi, 2008; Gu et al., 2009). MGAT3 and bisecting GlcNAc structures participate in processes such as differentiation and carcinogenesis by regulating functions of target glycoproteins. In previous studies, MGAT3 expression was positively regulated by E-cadherin/ β-catenin-mediated signaling, and negatively regulated by Wnt/ β-catenin signaling (Iijima et al., 2006; Akama et al., 2008; Xu et al., 2011, 2012). MGAT3 modified N-glycosylation of epithelial growth factor receptor (EGFR) and integrin, and inhibited cell surface binding of EGFR/ integrin to galectin-3, resulting in their endocytosis, suppression of intracellular signaling, and consequent promotion of cell migration and tumor metastasis (Partridge et al., 2004).

In the present study, we investigated (i) levels of bisecting GlcNAc structures and their inhibitory effects during hypoxia-induced EMT in breast cancer cell lines, using high-throughtput techniques (mass spectrometry, lectin microarray), and (ii) effects of MGAT3 on cell proliferation, migration, colony formation, and signaling pathways. Hypoxia as a common feature of most tumors, contributes to chemoresistance, radioresistance, angiogenesis, vasculogenesis, invasiveness, and metastasis. Tumor hypoxia might be considered the best validated target. Our findings showed that bisecting GlcNAc structures retarded the EMT progression induced by hypoxia, which suggested the therapeutic potential of enhancing MGAT3 as a treatment for controlling breast cancer development.

## Materials and methods

### Cell lines and culture

Human breast cancer MCF7 and MDA-MB-231 cell lines were from American Type Culture Collection (ATCC; Manassas, VA, USA). Cells were cultured in DMEM supplemented with 10% fetal bovine serum (Biological Industries; Kibbutz Beit Haemek, Israel) and 1% penicillin-streptomycin (Gibco; Carlsbad, CA, USA) at 37°C in 5% CO_2_ atmosphere as described in other study (Mertens-Talcott et al., 2007; Kim et al., 2010; Wardi et al., 2014). For hypoxic treatment, cells were maintained in an *in vivo* 200 hypoxia chamber (Ruskinn; Bridgend, UK) with 1% O_2_/ 5% CO_2_/ 94% N_2_ atmosphere for 24 h as previously described(Nagpal et al., 2015; He et al., 2017).

### Total protein extraction

Total proteins were extracted with T-PER Reagent (Thermo Scientific; San Jose, CA) as described previously (Tan et al., 2014). In brief, cells (~1 × 10^7^) were detached with trypsin, washed twice with ice-cold 1 × PBS (0.01 M phosphate buffer containing 0.15 M NaCl, pH 7.4), lysed with 1 mL T-PER Reagent containing protease inhibitor cocktail (Sigma-Aldrich; St. Louis, MO, USA) and phosphatase inhibitor cocktail (Sigma-Aldrich), incubated for 30 min on ice, homogenized, and centrifuged at 12,000 rpm for 15 min. The supernatant was collected and stored at −80°C. Protein concentration was determined by BCA assay (Beyotime; Haimen, Jiangsu, China).

### Western blotting

Western blotting was performed as described previously (Tan et al., 2014). In brief, total proteins (30 μg) from normoxia- and hypoxia-treated samples were separated by 7.5% SDS-PAGE. Gels were transferred onto polyvinylidene difluoride (PVDF) membranes with Trans-Blot Turbo Transfer System (Bio-Rad; Hercules, CA). Membranes were soaked in 5% skim milk in TBST (20 mM Tris-HCl, 150 mM NaCl, 0.05% Tween 20, pH 8.0) for 2 h at 37°C, probed with primary antibodies against MGAT3 (1:500; ab135514; Abcam, Cambridge, MA, UK), fibronectin (1:1000; ab2413; Abcam), E-cadherin (1:10000; 610181; BD Biosciences, San Jose, CA, USA), β-catenin (1:5000; ab32572; Abcam), tubulin (1:5000; T7816; Sigma-Aldrich, St. Louis, MO, USA), HIF-1α (1:1000; 3716; Cell Signaling Technology, Beverly, MA, USA), GLUT1 (1:5000; ab40084; Abcam), AKT (pan) (1:1000; 4685; Cell Signaling Technology), and p-AKT (Ser473) (1:2000; 4060; Cell Signaling Technology) overnight at 4°C and incubated with appropriate HRP-conjugated secondary antibody. Specific bands were visualized with a Pro-light HRP Kit (Tiangen; Beijing, China).

### Wound healing assay

Wound healing assay was performed as described previously (Castro et al., 2018). Migratory capacity of cells was determined by wound healing assay. In brief, cells (2 × 10^5^ per well, in a six-well plate) were cultured overnight and treated as described above. Three separate wounds were scratched with a pipette tip on the cell monolayer in each well, moving perpendicularly to a line drawn at the bottom of the plate. Cells were rinsed twice with 1 × PBS twice, added with fresh serum-free medium, and wounds at marked lines were photographed. After 24 h incubation at 37°C under normoxia or hypoxia, cells were washed with ice-cold 1 × PBS, and wound tracks were photographed and marked using ImagePro Plus software (Media Cybernetics; Silver Spring, MD, USA).

### Cell proliferation

Cell proliferation was performed as described previously (Yu et al., 2008). Cells were plated in 96-well plates, and incubated 4 h with CellTiter 96 AQ_ueous_. One Solution Cell Proliferation Assay (MTS) solution (Promega; Madison, WI, USA). MTS products in supernatant were transferred into 96-well microtiter plates, and absorbance at 490 nm was determined.

### Colony formation

Colony formation was performed as described previously(Yu et al., 2008). Cells (2,500 per well) were plated in a 6-cm dish, and grown 1–2 weeks until small colonies were clearly seen. Medium was discarded, cells were rinsed twice with 1 × PBS, fixed with 2% fresh paraformaldehyde, stained with crystal violet solution, and photos were taken. 10% acetic acid solution (1 mL) was added to dissolve the crystal violet. Optical density at 595 nm (OD 595) was measured.

### RNA isolation

RNA isolation was performed as described previously (Tan et al., 2014). Cells (1 × 10^5^ per well in a six-well plate) were cultured and treated as described above. Total RNA was isolated using an RNApure Tissue Kit (CWBiotech; Beijing) as per the manufacturer's instructions.

### Quantitative real-time PCR

Real-time PCR was performed as described previously (Tan et al., 2014). Total RNA was extracted as above. Primers were selected from qPrimerDepot (primerdepot.nci.nih.gov/) (Cui et al., 2007). First-strand cDNA was synthesized from total RNA using a ReverTra Ace-α First-strand cDNA Synthesis Kit (Toyobo; Osaka, Japan). Quantitative real-time PCR was performed by LightCycler-based SYBR Green I dye detection with UltraSYBR Mixture (CWBiotech). Gene expression was quantified by the 2^−ΔΔCT^ method (Livak and Schmittgen, 2001).

### Lectin microarray analysis

Lectin microarrays were constructed and analyzed as described previously (Qin et al., 2012; Yu et al., 2012). In brief, 37 commercial lectins from Vector Laboratories (Burlingame, CA), Sigma-Aldrich, and Calbiochem Merck (Darmstadt, Germany) were immobilized onto a solid support at high spatial density. Glycoprotein samples labeled with fluorescent dye Cy3 (GE Healthcare; Buckinghamshire, UK) were applied to the lectin microarrays, and the arrays were scanned with a GenePix 4000B confocal scanner (Axon Instruments; Union City, CA).

### Lectin staining

Lectin staining was performed as described previously (Tan et al., 2014). Cells were cultured in 24-well plates with sterilized coverslips to obtain monolayers with 70–80% confluence. Cells were washed with ice-cold 1 × PBS, immobilized with 2% fresh paraformaldehyde for 15 min at room temperature (RT), permeabilized with 0.2% Triton X-100 in 1 × PBS for 10 min at RT, and blocked with 5% BSA in 1 × PBS for 1 h at 37°C. Fixed cells were incubated with 15–20 μg/mL Cy3 fluorescein-labeled lectins (Con A, MAL-I, LCA, PHA-E) in 5% BSA for 3 h in the dark at RT, washed with 1 × PBS, stained with 20 μg/mL DAPI in 1 × PBS for 10 min at RT, washed again with 1 × PBS, and photographed with a fluorescence microscope (model Eclipse E600; Nikon; Tokyo, Japan).

### N-glycan separation

N-glycans were separated as described previously (Tan et al., 2014). In brief, total proteins (2 mg) from each cell line were concentrated and desalted using a size-exclusion spin ultrafiltration unit (Amicon Ultra-0.5 10 KD, Millipore; Billerica, MA). Proteins were denatured with 8 M urea, 10 mM DTT, and 10 mM IAM (Sigma-Aldrich) and centrifuged. The sample was further digested with PNGase F (New England BioLabs; Ipswich, MA) overnight at 37°C. Released N-glycans were collected and lyophilized.

### Desalting of N-glycans

N-glycans were desalted as described previously (Tan et al., 2014). N-glycans were desalted using Sepharose 4B (Sigma-Aldrich) as described previously (Tan et al., 2014). In brief, Sepharose 4B in a microtube was pre-equilibrated with methanol/ H_2_O (1:1 v/v) (MW) and 1-butanol/ methanol/ H_2_O (5:1:1 v/v/v) (BMW). Glycans were dissolved in 500 μL BMW, added to the Sepharose 4B, and the mixture was shaken gently and washed with BMW. N-glycans were eluted with MW, and the eluent was collected and lyophilized.

### Mass spectrometry

Mass spectrometry analysis of N-glycans were performed as described previously (Tan et al., 2014). N-glycans were characterized by MALDI-TOF-MS (UltrafleXtreme, Bruker Daltonics; Bremen, Germany). Lyophilized N-glycans were resuspended in 10 μL MW, and 1 μL of the mixture was spotted onto an MTP AnchorChip sample target and air-dried. 1 μL of 20 mg/mL 2, 5-dihydroxybenzoic acid (DHB) in MW was spotted to recrystallize the glycans. Measurements were taken in positive-ion mode, and m/z data were analyzed and N-glycan structures were annotated using the GlycoWorkbench software program (code. google.com/p/glycoworkbench/). Relative intensity was analyzed and generated using FlexAnalysis software (Bruker Daltonics) based on MALDI-TOF-MS intensity. Relative proportion was calculated by accumulating the relative intensity of a given type N-glycan.

### Overexpression and knockdown of MGAT3 in MCF7 cells

MGAT3 was amplified via PCR and linked to lentiviral overexpression vector pLVX-AcGFP1-N1 (Takara; Shiga, Japan). Lentiviral shRNA vector is constructed based on pLVX-shRNA2-Puro (Takara). Target sequences are listed below. Lentiviral vectors were packed in HEK293T via the packaging system, together with pMD2.G and psPAX2 (Addgene; Cambridge, MA, USA). Transfected MCF7 cells were selected and enriched by adding puromycin to culture medium.

**Table:** 

Sequence	
Target1	TGTATGGGCTGGACGGCAT
Target2	CCCAACTTCAGACAGTATGA

### Statistical analysis

Data are presented as mean ± *SD*. Statistical significance of differences between means was evaluated by Student's *t*-test at *p* < 0.05.

## Results

### Hypoxia induces EMT and increases migration in hypoxia-treated cells

**Figure 1:**
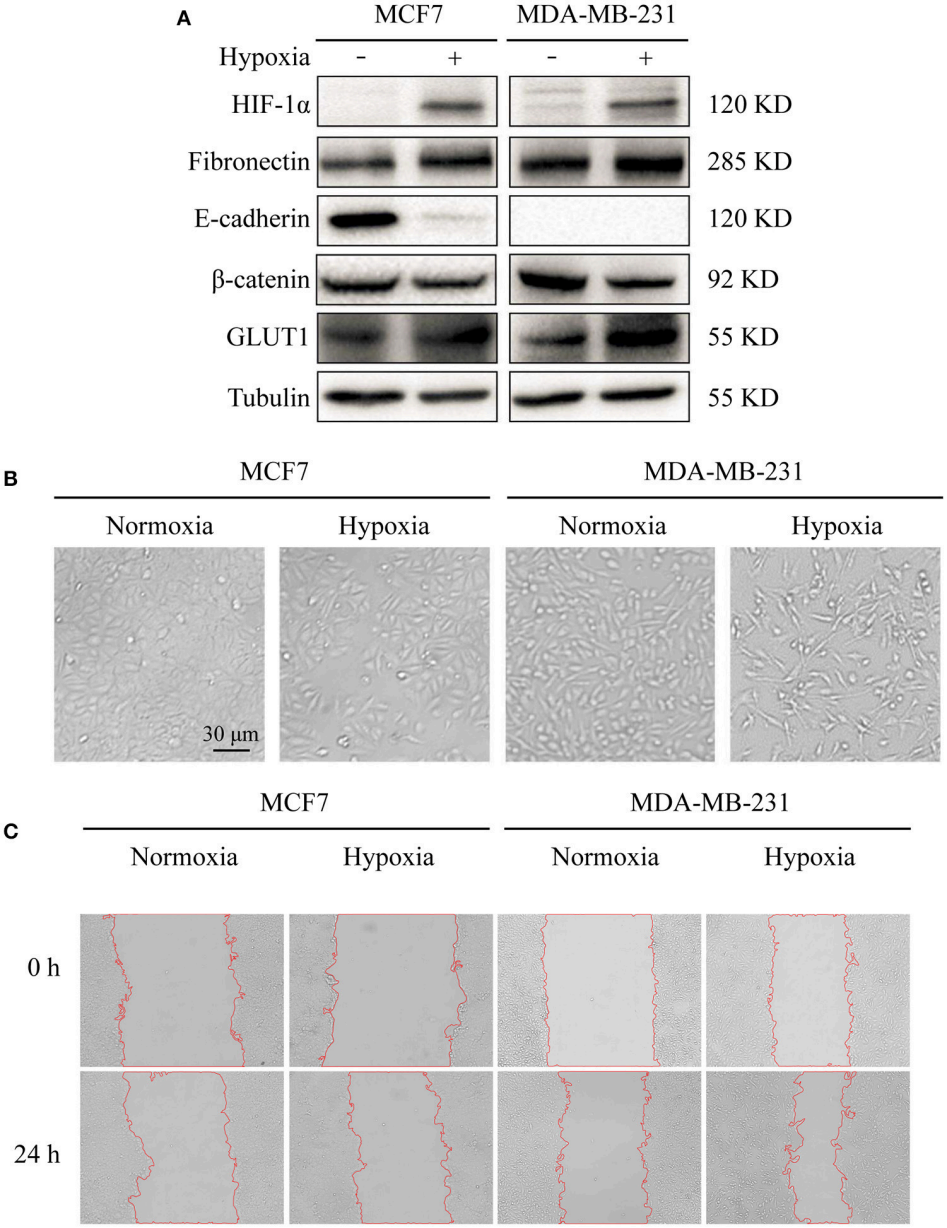
Effects of hypoxia on cell markers, morphology, and migration **(A)** Expression in breast cancer MCF7 and MDA-MB-231 cells of E-cadherin (epithelial marker), fibronectin (epithelial marker), HIF-1α (hypoxia marker), β-catenin, and GLUT1. Cells were cultured at 37°C in 5% CO_2_ atmosphere for normoxic treatment, and in 1% O_2_/ 5% CO_2_/ 94% N_2_ atmosphere for hypoxic treatment. Cells were harvested, lysed in T-PER Reagent, and protein content was determined by BCA assay. Western blotting was performed as described in M&M. **(B)** Morphological changes under normoxic and hypoxic conditions. Cells (2 × 10^5^ per well) were grown in 6-well plates for 24 h under the two conditions. Photos were taken by phase-contrast microscopy at 200× magnification. **(C)** Cell migration assessed by wound assay. Cell monolayers under the two conditions were scratched with pipette tip. Cells were washed with ice-cold 1× PBS and cultured in serum-free medium. Pictures of wounds were taken at 0 and 24 h by phase-contrast microscopy (100× magnification).

Previous studies using various cancer cell models have demonstrated induction of EMT by hypoxic conditions (Zhou et al., 2009; Copple, 2010). In the present study, we exposed breast cancer cell lines MCF7 and MDA-MB-231 to hypoxic environments. HIF-1α, an indicator of hypoxic conditions, was strongly expressed in hypoxia-treated cells (Figure 1A). The treated cells also showed loss of an epithelial character (E-cadherin) and acquisition of a mesenchymal character (fibronectin), typical hallmarks of EMT, and elevated expression of glucose transporter-1 protein (GLUT1), a putative intrinsic cellular marker of hypoxia regulated by HIF-1α (Figure 1A).

Hypoxia treatment (1% O_2_) of the two cell lines for 72 h resulted in altered morphology in comparison with normoxia (21% O_2_) treated cells. Hypoxia-treated cells showed elongation and flattening (Figure 1B), and strongly enhanced migratory capacity, particularly in MCF7 (Figure 1C).

### N-glycan profiles of normoxia- and hypoxia-treated cells

**Figure 2:**
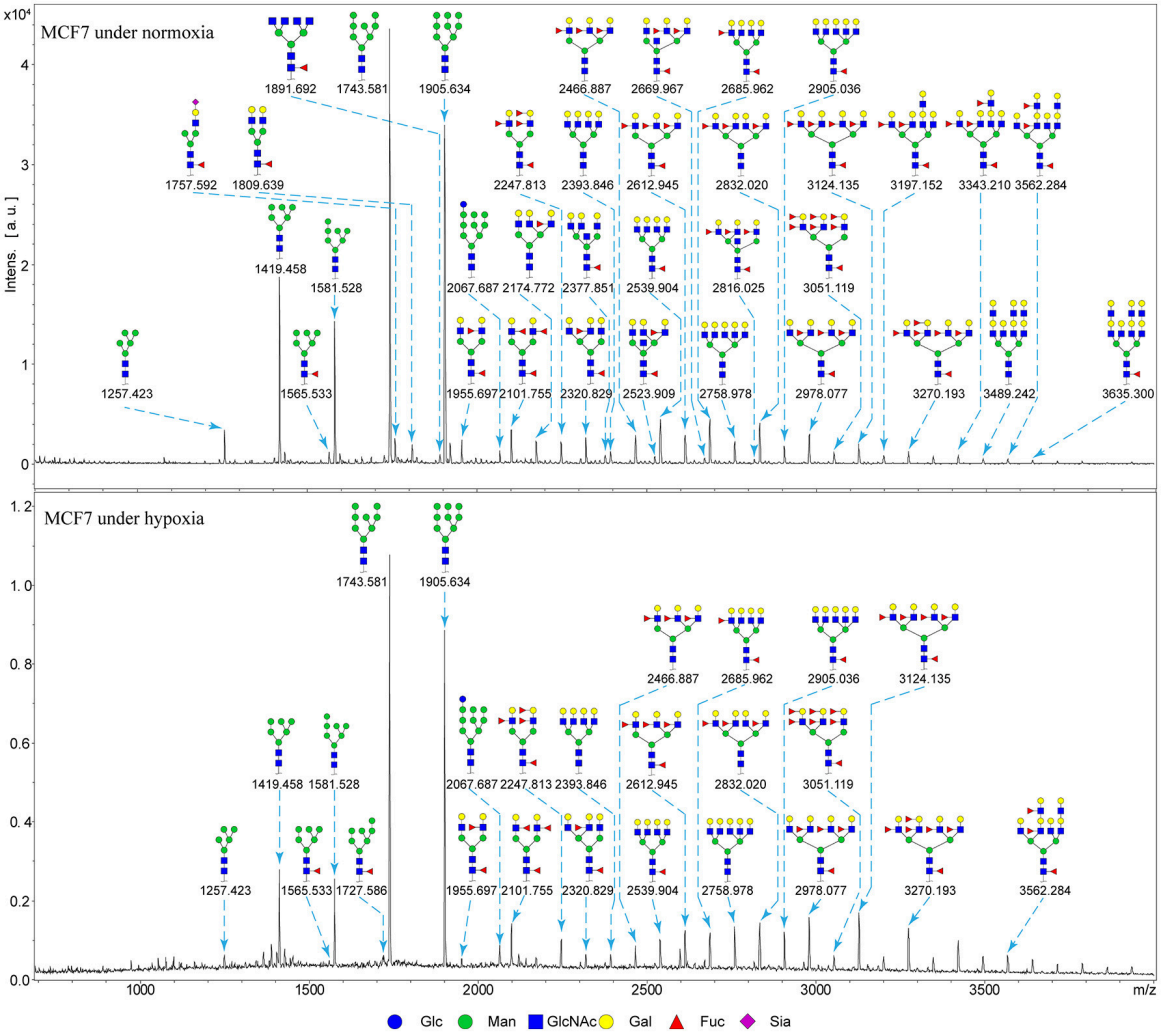
MALDI-TOF-MS spectra of N-glycans from MCF7 cells MCF7 cells were cultured in 10-cm dishes under normoxic and hypoxic conditions, and N-glycans were separated and desalted as described in M&M. Lyophilized N-glycans were dissolved in MW, and an aliquot of mixture with DHB solution was spotted on MTP AnchorChip sample target and air-dried. MALTI-TOF-MS was performed in positive-ion mode. Experiments were performed in biological triplicate, and representative N-glycan spectra are shown. Peaks (signal-to-noise ratio > 5) were selected for relative proportion analysis. Detailed structures were analyzed using the GlycoWorkbench program. Proposed structures are indicated by m/z value.

**Figure 3:**
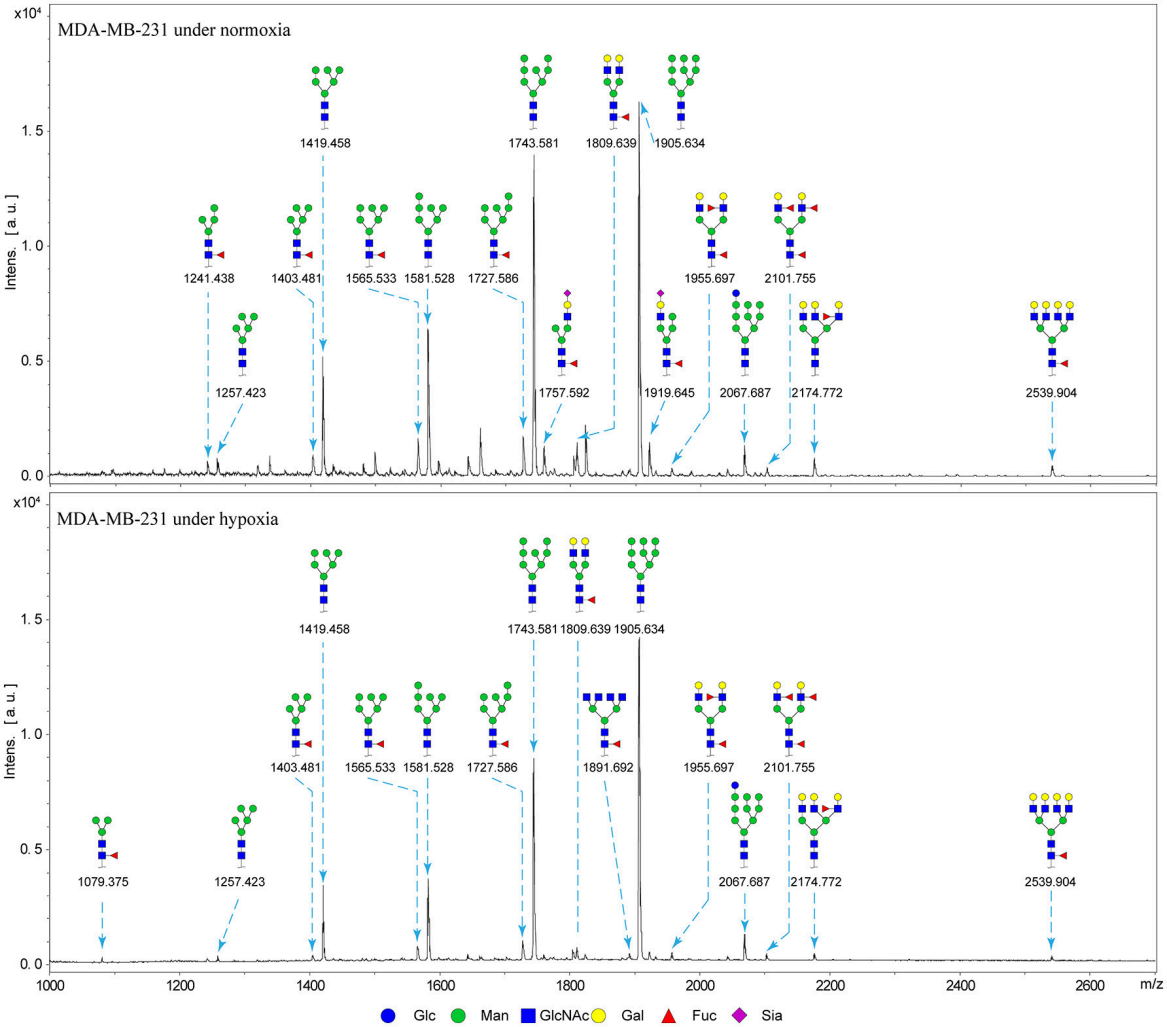
MALDI-TOF-MS spectra of N-glycans from MDA-MB-231 cells MDA-MB-231 cells were cultured in 10-cm dishes under normoxic and hypoxic conditions, and N-glycans were separated and desalted as described in M&M. Lyophilized N-glycans were dissolved in MW, and an aliquot of mixture with DHB solution was spotted on MTP AnchorChip sample target and air-dried. MALTI-TOF-MS was performed in positive-ion mode. Experiments were performed in biological triplicate, and representative N-glycan spectra are shown. Peaks (signal-to-noise ratio > 5) were selected for relative proportion analysis. Detailed structures were analyzed using the GlycoWorkbench program. Proposed structures are indicated by m/z value.

Alterations in glycosylation have been observed in numerous physiological and pathological processes. To identify specific N-glycan alterations during hypoxia, we used MALDI-TOF-MS to compare glycomic profiles of normoxia- vs. hypoxia-treated cells. Compositional and structural features were indicated in representative MALDI-TOF-MS spectra with signal-to-noise ratios >5 (Figures 2, 3, Tables S1, S2; Supplementary Information).

Thirty-four individual N-glycan masses were detected in MCF7, and 19 in MDA-MB-231. Numbers of distinct N-glycans were: 33 in normoxia-treated MCF7, 25 in hypoxia-treated MCF7, 17 in normoxia-treated MDA-MB-231, and 16 in hypoxia-treated MDA-MB-231. N-glycan structures found exclusively under hypoxic conditions in each of the two cell lines were mostly complex or hybrid type rather than high-mannose type.

We examined quantitative differences in various N-glycan types under the two conditions, as shown by the relative proportions summarized in Table 1. Proportions of bisecting GlcNAc structures were reduced under hypoxia vs. normoxia (2.51 vs. 5.35% in MCF7; 3.62 vs. 5.65% in MDA-MB-231). Proportions under hypoxia vs. normoxia were also reduced for hybrid type N-glycans (2.94 vs. 4.34% in MCF7; 1.47 vs. 3.66% in MDA-MB-231) and for sialylation (0.43 vs. 0.76% in MCF7; 0.00 vs. 3.02% in MDA-MB-231). Proportions for fucosylation under hypoxia vs. normoxia were elevated in MCF7 (22.57 vs. 17.03%) but reduced in MDA-MB-231 (13.58 vs. 21.62%). Detailed information regarding substituents and branching patterns of N-glycans was obtained by MALDI-TOF/TOF-MS/MS (Figure S1).

**Table 1:** Relative proportions of various types of N-glycans in MCF7 and MDA-MB-231 cells under normoxia and hypoxia.

**Glycan type**	**Relative proportion (%) of MCF7**		**Relative proportion (%) of MDA-MB-231**	
	**Normoxia**	**Hypoxia**	**Normoxia**	**Hypoxia**
High-mannose	83.52 ± 0.54	77.91 ± 11.07	89.98 ± 2.21	95.10 ± 1.00
Hybrid	4.34 ± 0.54	2.94 ± 0.73	3.66 ± 1.34	1.47 ± 0.27
Complex	15.67 ± 0.57	21.46 ± 9.98	8.63 ± 1.00	4.90 ± 1.00
Multi-antennary	16.48 ± 0.54	22.09 ± 11.07	7.00 ± 0.41	4.90 ± 1.00
Bisecting GlcNAc	5.35 ± 0.11	2.51 ± 0.04	5.65 ± 1.23	3.62 ± 0.66
Sialylation	0.76 ± 0.58	0.43 ± 0.75	3.02 ± 2.61	0.00
Fucosylation	17.03 ± 0.19	22.57 ± 8.31	21.62 ± 3.35	13.58 ± 1.54

### Glycopattern alteration in hypoxia-treated cells

**Figure 4:**
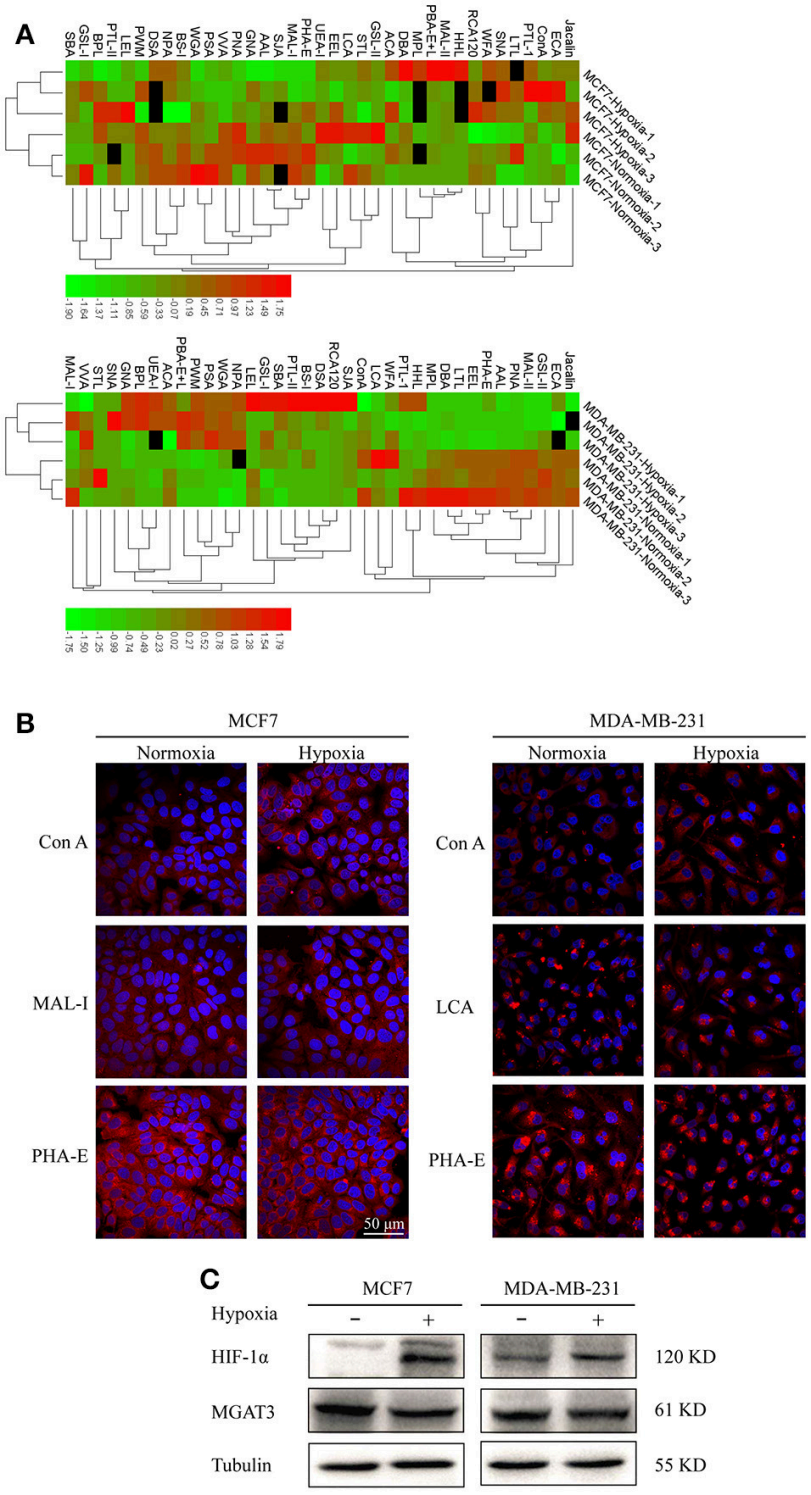
Variation of fine glycan structures detected by lectin microarray analysis **(A)** Variation of levels of glycans from MCF7 (upper) and MDA-MB-231 (lower) cells, detected by 37 lectins, is presented as a heatmap. Lectin microarray analysis was performed as described as M&M. Red: fluorescence signal activation. Green: signal inhibition. Black: missing data. **(B)** Altered glycan levels evaluated by lectin histochemistry. Four lectins (Con A, MAL-I, LCA, PHA-E) were applied, and lectin histochemistry was performed as described in M&M. Signals are shown from merge images of Cy3-conjugated lectins and DAPI staining of nuclei in MCF7 (left) and MDA-MB-231 (right) under normoxic and hypoxic conditions (60× magnification). **(C)** Expression in MCF7 and MDA-MB-231 cells of HIF-1α, MGAT3, and tubulin.

Lectin microarray analysis was used to identify specific glycopatterns in hypoxia-treated vs. normoxia-treated cells. The microarray was used to analyze fine glycan structures of glycoproteins in cells under the two conditions contained 37 lectins (Table S3), including two negative controls (BSA) and one positive control (Cy3-BSA). Under hypoxia, significant differences (>1.5-fold or <0.67-fold) were observed for glycans recognized by 12 different lectins in MCF7 and by 22 different lectins in MDA-MB-231 (Figure 4A). In both cell lines, bisecting GlcNAc structures (recognized by PHA-E), core fucosylation (recognized by PSA), and T antigen (recognized by PNA) were significantly suppressed, whereas non-substituted α1,6-Man structures (recognized by HHL) was elevated (Tables 2, 3). A heatmap produced by complete hierarchical clustering and visualization using the HemI 1.0 software program (hemi.biocuckoo.org/down.php) (Deng et al., 2014) showed side-by-side clustering of normoxia- and hypoxia-treated cells in the dendrogram for both MCF7 and MDA-MB-231 (Figure 4A).

**Table 2:** Differential glycopatterns in normoxia- vs. hypoxia-treated MCF7 cells revealed by lectin microarray analysis.

**Lectin**	**Abbreviation**	**Specificity**	**FC of MCF7 (H/N)**
*Maackia amurensis* lectin I	MAL-I	Galβ-1,4GlcNAc 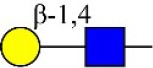	0.45
*Pisum sativum* agglutinin	PSA	Fucα-N-acetylchitobiose-Man 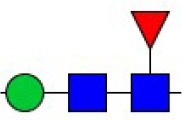	0.46
*Phaseolus vularis* erythroagglutinin	PHA-E	Bisecting GlcNAc 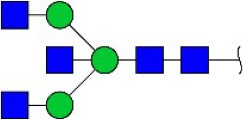	0.60
Peanut agglutinin	PNA	Galβ1-3GalNAcα-Ser/Thr(T) 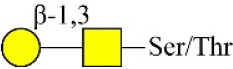	0.60
*Lotus tetragonolobus* lectin	LTL	Fucα-1,3GlcNAc (core) 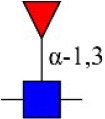	1.61
*Concanavalin ensiformis* agglutinin	Con A	Branched and terminal Man 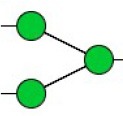	1.61
Soybean agglutinin	SBA	Terminal GalNAc (especially GalNAcα1-3Gal) 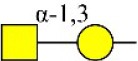	1.74
*Maclura pomifera* lectin	MPL	αGalNAc 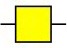	6.65
*Phaseolus vulgaris* agglutinin	PHA-E+L	Bisecting GlcNAc and β-1,6-GlcNAc 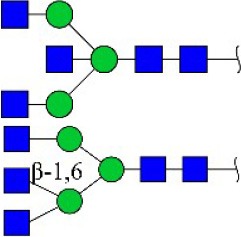	23.53
*Hippeastrum hybrid* lectin	HHL	Non-substituted α-1,6 Man 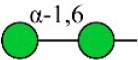	54.63
*Maackia amurensis* lectin II	MAL-II	Siaα2-3Galβ1-4Glc(NAc) 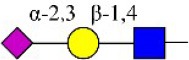	∞
*Psophocarpus tetragonolobus* lectin I	PTL-I	αGalNAc and Gal 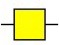 and 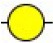	∞

**Table 3:** Differential glycopatterns in normoxia- vs. hypoxia-treated MDA-MB-231 cells revealed by lectin microarray analysis.

**Lectin**	**Abbreviation**	**Specificity**	**FC of MDA-MB-231 (H/N)**
*Maackia amurensis* lectin I	GSL-II	Galβ-1,4GlcNAc 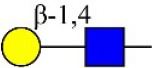	0.00
Peanut agglutinin	PNA	Galβ1-3GalNAcα-Ser/Thr(T) 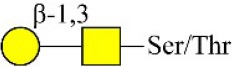	0.00
Soybean agglutinin	SBA	Terminal GalNAc (especially GalNAcα1-3Gal) 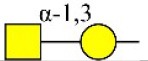	0.00
*Wistera floribunda* agglutinin	WFA	GalNAcα/β1-3/6Gal 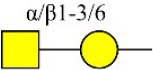	0.02
*Bandeiraea simplicifolia* lectin-I	BS-I	α-Gal, α-GalNAc 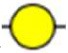 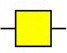	0.06
*Pisum sativum* agglutinin	PSA	Fucα-N-acetylchitobiose-Man 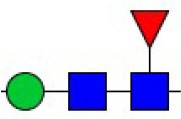	0.09
Concanavalin A	Con A	Branched and terminal Man 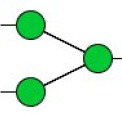	0.27
*Aleuria aurantia* lectin	AAL	Terminal Fucα-1,6GlcNAc 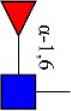 Fucα-1,3Galβ-1,4GlcNAc 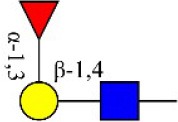	0.32
*Maackia amurensis* lectin II	MAL-II	Siaα2-3Galβ1-4Glc(NAc) 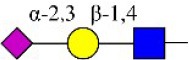	0.34
*Lens culinaris* agglutinin	LCA	Fucα-1,6GlcNAc (core) 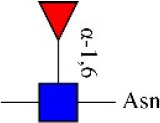	0.48
*Erythrina cristagalli* agglutinin	ECA	Galβ-1,4GlcNAc 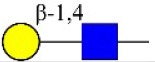	0.56
*Psophocarpus tetragonolobus* lectin I	PTL-I	αGalNAc and Gal 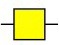 and 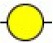	0.60
*Phaseolus vularis* erythroagglutinin	PHA-E	Bisecting GlcNAc 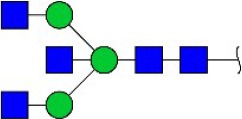	0.60
*Narcissus pseudonarcissus* agglutinin	NPA	Non-substituted α-1,6 Man 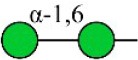	1.76
*Vicia villosa* agglutinin	VVA	GalNAcα-Ser/Thr(Tn) 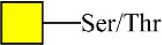	1.87
Jacalin	Jacalin	Galβ1-3GalNAcα-Ser/Thr(TF) 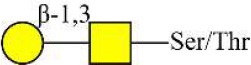 GalNAcα-Ser/Thr(T) 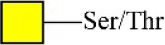	2.11
*Datura stramonium* agglutinin	DSA	GlcNAc 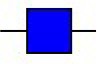	2.33
*Solanum tuberosum* lectin	STL	(GlcNAc)_n_ 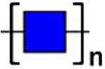	128.47
*Griffonia simplicifolia*-lectin-I	GSL-I	αGalNAc, αGal 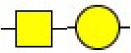 GalNAcα-Ser/Thr(Tn) 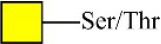	156.70
*Sophora japonica* agglutinin	SJA	Terminal GalNAc and Gal 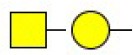	175.95
*Hippeastrum hybrid* lectin	HHL	Non-substituted α-1,6 Man 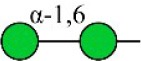	251.86
*Euonymus europaeus* lectin	EEL	Galα1-3(Fucα1-2)Gal 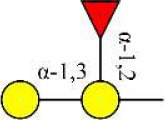	∞

Glycan profiles under hypoxic conditions were further investigated and confirmed by histochemistry using PHA-E, MAL-I, LCA, and Con A (Figure 4B). Both MCF7 and MDA-MB-231 showed strong reduction of PHA-E and increase of Con A fluorescence signals. MCF7 showed reduction of MAL-I and MDA-MB-231 showed reduction of LCA signals, consistently with results of lectin microarray analysis. On the other hand, MDA-MB-231 microarray results using Con A were inconsistent with the elevated high-mannose N-glycan levels detected by mass spectrometry, lectin histochemistry, and microarray results using NPA and HHL. This apparent discrepancy may be due to random orientation of Con A following immobilization on glass slides (Qin et al., 2012). We examined expression of MGAT3 (which catalyzes synthesis of bisecting GlcNAc structures) in hypoxia-treated MCF7 and MDA-MB-231, and found that it was decreased at protein level (Figure 4C).

### Overexpression of MGAT3 inhibits hypoxia-induced EMT in MCF7 cells

**Figure 5:**
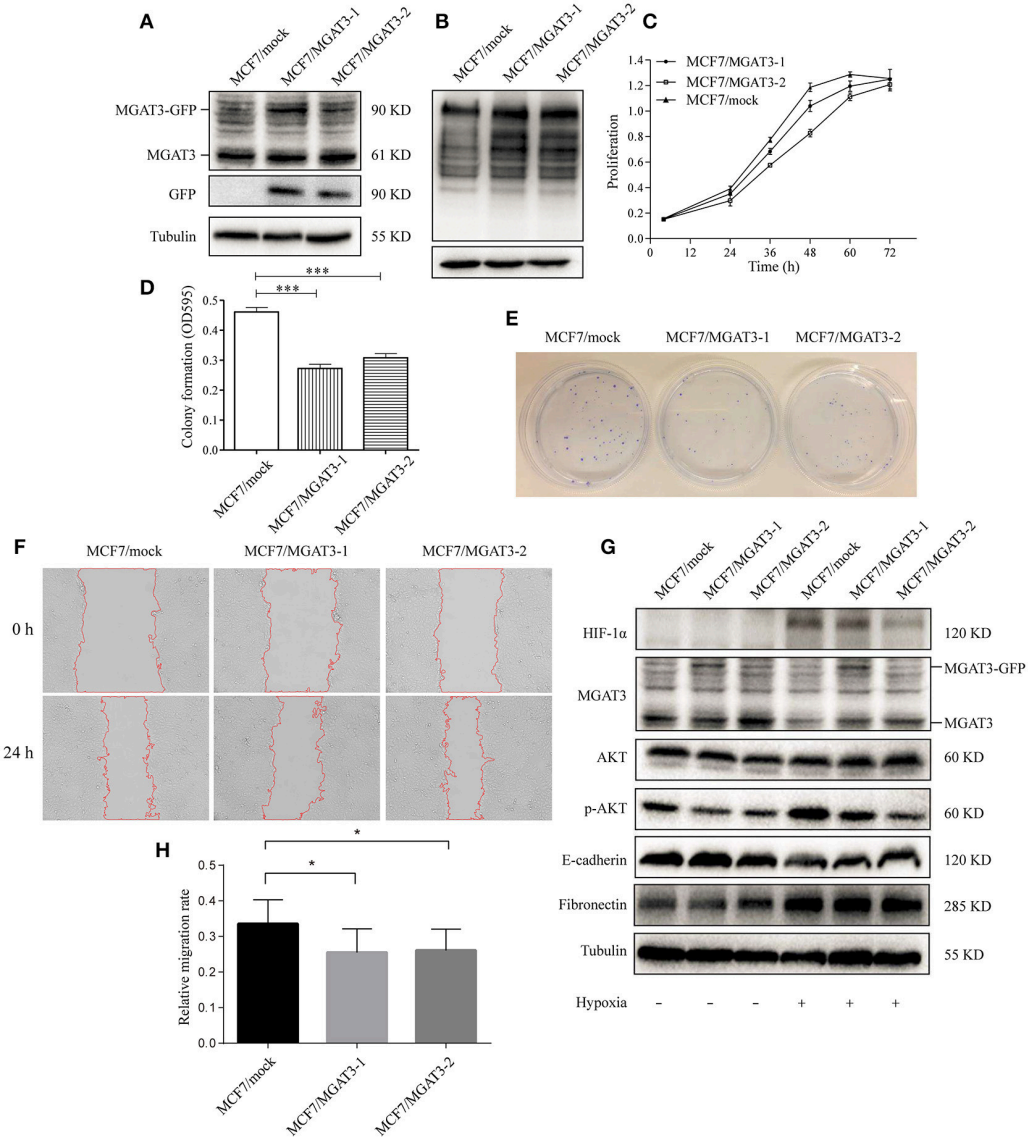
MGAT3 overexpression suppresses hypoxia-induced EMT in MCF7 cells **(A)** MGAT3 expression in mock- and MGAT3-transfected MCF7 cells. Cells were stably transduced with a GFP-marked lentivirus carrying mock gene or MGAT3 gene, harvested, and lysed in T-PER Reagent. Western blotting was performed as described in M&M using anti-MGAT3 and anti-GFP antibody. **(B)** Levels of bisecting GlcNAc structures in mock- and MGAT3-transfectants. Whole cell lysates of the two transfectants were subjected to PHA-E lectin blotting as described in M&M. **(C)** Proliferation of transfectant cells. The two transfectants were cultured for 24, 36, 48, 60, and 72 h, and proliferation was assessed by MTS assay. **(E)** Colony formation ability. The two transfectants (2500 cells each) were cultured in 6-cm dishes for 1–2 week, fixed, stained with crystal violet solution, and photographed. Acetic acid was added to dissolve crystal violet, and OD 595 was determined **(D)**. ^*^p < 0.05; ^***^p < 0.001. **(F)** Cell migration. Migration assays of the two transfectants under normoxic and hypoxic conditions were performed as described in M&M, and relative migration rate was shown **(H)**. ^*^p < 0.05. **(G)** Expression of HIF-1α, MGAT3, AKT, p-AKT, E-cadherin, fibronectin, and tubulin in the two transfectants under normoxic and hypoxic conditions. Cells were cultured as described in Figure 1, harvested, and lysed in T-PER Reagent. Protein content was determined by BCA assay. Western blotting was performed as described in M&M.

To elucidate the functional effects of reduced levels of bisecting GlcNAc structures and MGAT3 during hypoxia, we overexpressed *MGAT3* gene in MCF7. Cells were stably transduced with a green fluorescent protein (GFP)-marked lentivirus carrying mock (MCF7/mock) or *MGAT3* (MCF7/MGAT3-1/2). *MGAT3* overexpression was confirmed by western blotting (Figure 5A) and PHA-E lectin blotting (Figure 5B), and resulted in increased expression of MGAT3 and bisecting GlcNAc structures. The elevation of MGAT3 level inhibited proliferation (Figure 5C), colony formation (Figures 5D,E), and migration (Figures 5F,H) of the cells.

Previous studies have shown that MGAT3 regulates activation of signaling pathways such as extracellular signal-regulated kinase (ERK) 1/2 or protein kinase B (AKT) (Miwa et al., 2013), and is involved in EMT and its reversed process, mesenchymal-epithelial transition (MET) (Pinho et al., 2012; Xu et al., 2012). We therefore examined possible effects of aberrant MGAT3 expression on relevant signaling pathway during hypoxia. In mock transfectants under hypoxia, reduced E-cadherin expression, increased fibronectin expression, and activation of AKT signaling were observed (Figure 5G). In MGAT3 transfectants under hypoxia, MGAT3 overexpression had significant blocking effects on downregulation of E-cadherin expression, upregulation of HIF-1α expression, and AKT signaling activation, and a slight blocking effect on upregulation of fibronectin expression (Figure 5G). These findings, taken together, indicate that MGAT3 overexpression inhibits hypoxia-induced EMT.

### MGAT3 knockdown promote hypoxia-induced EMT in MCF7

**Figure 6:**
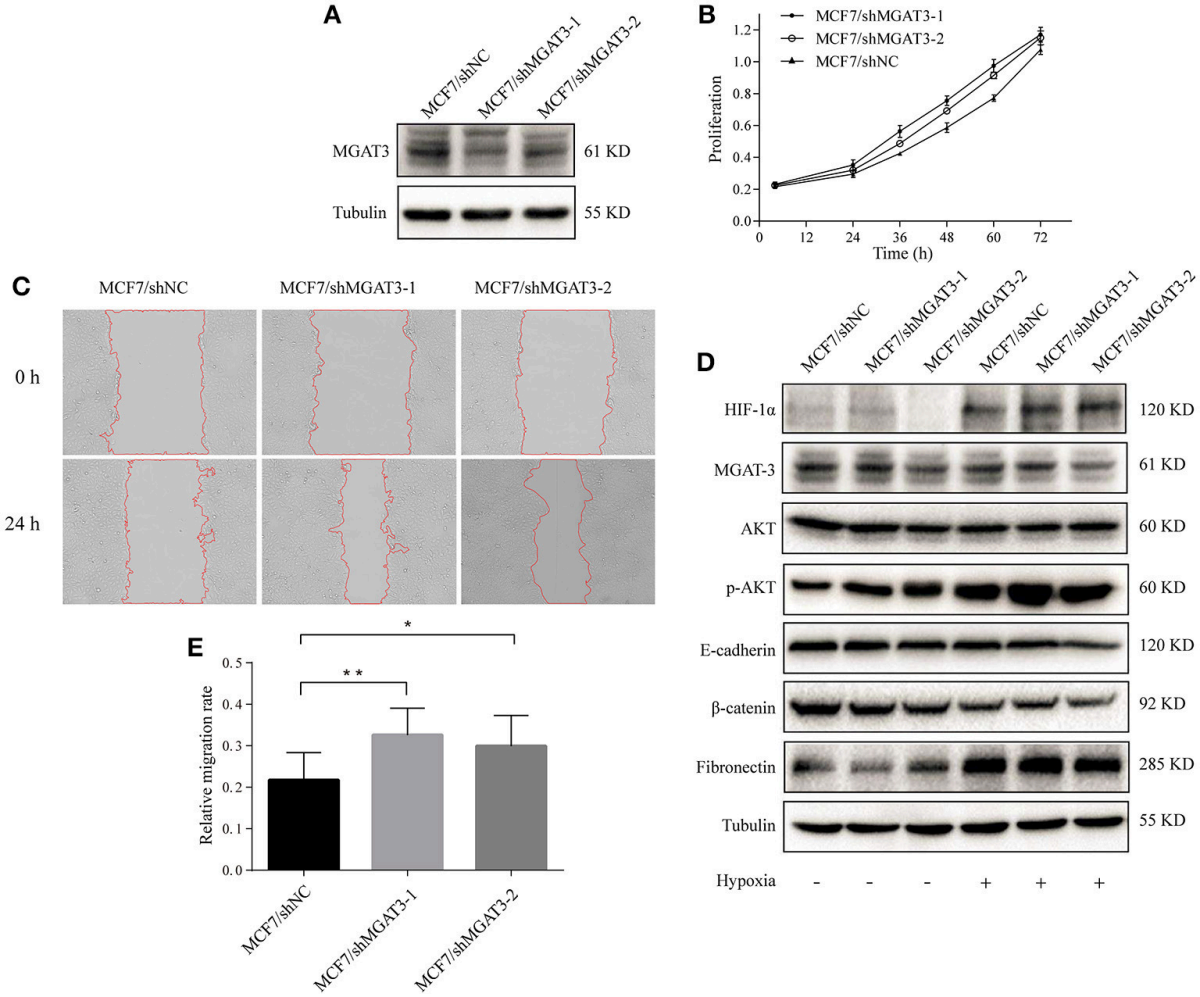
MGAT3 knockdown promotes hypoxia-induced EMT in MCF7 cells **(A)** MGAT3 expression in mock- and MGAT3-shRNA-transfected MCF7 cells. Cells were stably transduced with lentivirus carrying anti-MGAT3 shRNAs (MCF7/shMGAT3-1/2) or shNC (MCF7/mock), harvested, and lysed in T-PER Reagent. Western blotting was performed as described in M&M. **(B)** Cell proliferation. The two transfectants were cultured for 24, 36, 48, 60, and 72 h, and proliferation was assessed by MTS assay. **(C)** Cell migration. Migration assays of the two transfectants under normoxic and hypoxic conditions were performed as described in M&M, and relative migration rate was shown **(E)**. ^*^p < 0.05; ^**^p < 0.01. **(D)** Expression of HIF-1α, MGAT3, AKT, p-AKT, E-cadherin, fibronectin, β-catenin, and tubulin in the two transfectants under normoxic and hypoxic conditions. Cells were cultured as described in Figure 1, harvested, and lysed in T-PER Reagent. Protein content was determined by BCA assay. Western blotting was performed as described in M&M.

We silenced MGAT3 expression in MCF7 using MGAT3 shRNAs (MCF7/shMGAT3-1/2) or shNC (MCF7/shNC). MGAT3 knockdown was confirmed by western blotting (Figure 6A). Cell proliferation and migration were enhanced in MGAT3-shRNA transfectants (Figures 6B,C,E). Under hypoxia, MGAT3 knockdown in these cells promoted downregulation of E-cadherin expression and AKT signaling activation, but had no notable effect on β-catenin expression (Figure 6D).

## Discussion

Hypoxic regions, arising from irregular blood flow, chaotic vasculature, and/or poor oxygen diffusion, are present throughout solid cancer tissues (Jiang et al., 2011), Whereas hypoxia is toxic to normal cells, genetic and adaptive changes that occur in cancer cells allow them to survive and proliferate under hypoxic conditions. Such processes are the basis of malignant phenotype and aggressive tumor behavior. HIF-1/2 activation under hypoxic conditions in tumors is a key mechanism leading to tumor aggressiveness, metastasis promotion, and patient mortality (Harris, 2002; Semenza, 2002; Maxwell, 2005). Under hypoxic conditions, the tumor microenvironment activates major EMT-triggering pathways (e.g., TGFβ signaling, Notch signaling) that facilitate tumor growth and metastasis, and HIF modulates major transcription factors (e.g., TWIST, SNAIL, SLUG, SIP1, ZEB1) that regulate EMT (Jiang et al., 2011). Hypoxia and constitutive HIF expression were each shown to induce EMT (Higgins et al., 2008; Moen et al., 2009).

We used an integrated strategy (mass spectrometry in combination with lectin microarray analysis) to elucidate aberrant expression of N-glycans in breast cancer cells under hypoxia. Hypoxia-treated MCF7 cells showed reduced levels of bisecting GlcNAc structures and MGAT3 expression, and elevated levels of multi-antennary structures and fucosylation. In the process of N-glycan biosynthesis, the enzymes MGAT3 (GnT-III), GnT-IV (generating multi-antennary structures with β1-4-linked GlcNAc on Manα1-3 arm), GnT-V (generating multi-antennary structures with β1-6-linked GlcNAc on Manα1-6 arm), and FUT8 (generating core fucosylation) are able to utilize biantennary structures as substrates for N-glycan elongatation, and formation of N-glycan structures is determined by interplay among these enzymes; e.g., bisecting GlcNAc structures catalyzed by MGAT3 inhibit further catalytic activity by FUT8 and GnT-V (Taniguchi and Kizuka, 2015). Reduced levels of bisecting GlcNAc structures and MGAT3 expression may promote elevated levels of multi-antennary structures and fucosylation. In hypoxia-treated MDA-MB-231, we observed reduced levels of bisecting GlcNAc and multi-antennary structures, and increased levels of high-mannose structures. These changes may reflect a blocked step in N-glycan synthesis that results in incomplete glycosylation in these cells during hypoxia.

MGAT3 was transfected into MCF7 to clarify the functional role of this enzyme and its products (bisecting GlcNAc structures) during hypoxia. E-cadherin expression was significantly decreased in mock-transfected cells, whereas the rate of such decrease was much lower in MGAT3-transfected cells. In previous studies, MGAT3 overexpression blocked downregulation of E-cadherin expression during TGFβ-induced EMT (consistently with our findings), delayed E-cadherin turnover, and reduced E-cadherin release from the cell surface (Yoshimura et al., 1996; Xu et al., 2012).

In the present study, exogenous MGAT3 blocked upregulation of p-AKT expression during hypoxia (Figure 5G). Bisecting GlcNAc structures and MGAT3 were shown previously to modify N-glycosylation of target proteins and to inhibit or activate related signaling pathways. For example, attachment of bisecting GlcNAc structures to growth factor receptors slowed tumor progression by inhibiting activation of growth factor signaling (Song et al., 2010), and modification by MGAT3 of integrin N-glycosylation inhibited its ligand binding ability and consequent integrin-mediated signaling (Isaji et al., 2004). MGAT3-catalyzed addition of bisecting GlcNAc structures reduced levels of (poly)N-acetyllactosamine (LacNAc) structures on growth factor receptors by inhibiting GnT-V and GnT-IV. These changes suppressed binding of growth factor receptors to galectin-3, which forms a galectin lattice on the cell surface for clustering of glycoproteins with LacNAc, and promotes constitutive endocytosis of growth factor receptors, thereby inhibiting downstream signaling and consequent cell proliferation and survival through suppression of Erk and AKT signaling (Partridge et al., 2004; Lau et al., 2007; Miwa et al., 2013), consistently with our findings (Figure 5G). Suppression of AKT signaling may inhibit EMT, proliferation, and metastasis (Grille et al., 2003). Overexpression of GnT-III in highly metastatic melanoma cells was associated with reduced metastasis potential, and MGAT3-transfected melanoma cell performed significantly suppressed lung metastasis (Taniguchi and Kizuka, 2015), which were consistent with our data. Moreover, MGAT3^−/−^ mice overexpressing the polyomavirus middle T (PyMT) oncogene under the control of the mouse mammary tumor virus (MMTV) promoter exhibited an increased tumor burden, increased migration and early metastasis to lung (Song et al., 2010). These studies were consistent with our data.

In the present study, MGAT3 overexpression blocked elevation of HIF-1α expression during hypoxia (Figure 5G), possibly through inhibition of AKT signaling. HIF-1α expression may be regulated by PI3K/ PTEN/ AKT/ mTOR signaling (Zhong et al., 2000). Stimulation of Her2 activity by heregulin could also induce of HIF-1α expression attributing to the 5′-untranslated region of HIF-1α mRNA directing heregulin to express a heterologous protein (Laughner et al., 2001). HIF-1α may affect hypoxia-induced EMT through TGFβ signaling pathway by increasing SMAD3 mRNA levels and release of latent TGF-β2 (Zhang et al., 2003; Jiang et al., 2011). HIF-1 may also bind to and stabilize activated Notch, thereby affecting Notch signaling and hypoxia-induced EMT (Soares et al., 2004; Gustafsson et al., 2005; Jiang et al., 2011).

To further clarify the functional roles of bisecting GlcNAc structures and MGAT3 during hypoxia, we produced MGAT3-overexpressing and MGAT3 knockdown mutants of MCF7 cells. Under hypoxic conditions, MGAT3 overexpression significantly blocked downregulation of E-cadherin expression, activation of AKT signaling, upregulation of HIF-1α expression, and hypoxia-induced EMT. These observations were consistent with those from MGAT3 knockdown cells.

Hypoxia as a negative clinical prognostic indicator, is known to affect the response of solid malignancies to radiation and tumor oxygenation, and hypoxia is a strong predictor of both overall and disease-free survival. Our study indicated that MGAT3 might be a potential therapeutic target of hypoxic region of breast cancer. However, this study was deficient in molecular mechanism and *in vivo* experiments. Our ongoing studies include the exploration of the genetic and epigenetic bases of aberrant MGAT3 expression in breast cancer, and identification of target proteins bearing bisecting GlcNAc structures, and validation these results *in vivo* with the mouse model, and will clarify the detailed mechanism whereby these structures inhibit hypoxia-induced EMT in breast cancer cells.

## Author contributions

FG, XL, and ZT: Designed experiments; CW and ZT: Carried out experiments; ZT: Analyzed experiments results; FG, XL, CW, and ZT: Wrote the manuscript.

### Conflict of interest statement

The authors declare that the research was conducted in the absence of any commercial or financial relationships that could be construed as a potential conflict of interest. The handling editor is currently co-organizing a Research Topic with one of the authors FG, and confirms the absence of any other collaboration.
