# Supplementary material for: Bisecting N-Acetylglucosamine Structures Inhibit Hypoxia-Induced Epithelial-Mesenchymal Transition in Breast Cancer Cells
Source: Front Physiol. 2018 Mar 9;9:210. doi: 10.3389/fphys.2018.00210 (PMC5854678; doi:10.3389/fphys.2018.00210)
Supplement: Supplementary file 1 [file Table1.DOCX]

Supplementary Material

Bisecting GlcNAc structures inhibit hypoxia-induced epithelial-mesenchymal transition in breast cancer cells

Zengqi Tan^1^, Chenxing Wang^2^, Xiang Li^1,3^, Feng Guan^1^

^1^College of Life Science, Northwest University, Xi'an, Shaanxi, China.

^2^School of Biotechnology, Jiangnan University, Wuxi, Jiangsu, China.

^3^Wuxi Medical School, Jiangnan University, Wuxi, Jiangsu, China.

***Correspondence to:**Feng Guan, College of Life Science, Northwest University, 229 Taibai North Road, Xi’an, Shaanxi 710069, China. E-mail: guanfeng@nwu.edu.cn. Tel: +86-29-88303534

**Table of Contents:**

Fig. S1: MALDI-TOF/TOF-MS/MS analysis of N-glycan precursor ions in MS spectra.

Table S1. Proposed structures and their molecular ions of N-glycans from MCF7 cells under normoxia and hypoxia.

Table S2. Proposed structures and their molecular ions of N-glycans from MDA-MB-231 cells under normoxia and hypoxia.

Table S3: Glycan-binding specificities of the lectins used in lectin microarrays.


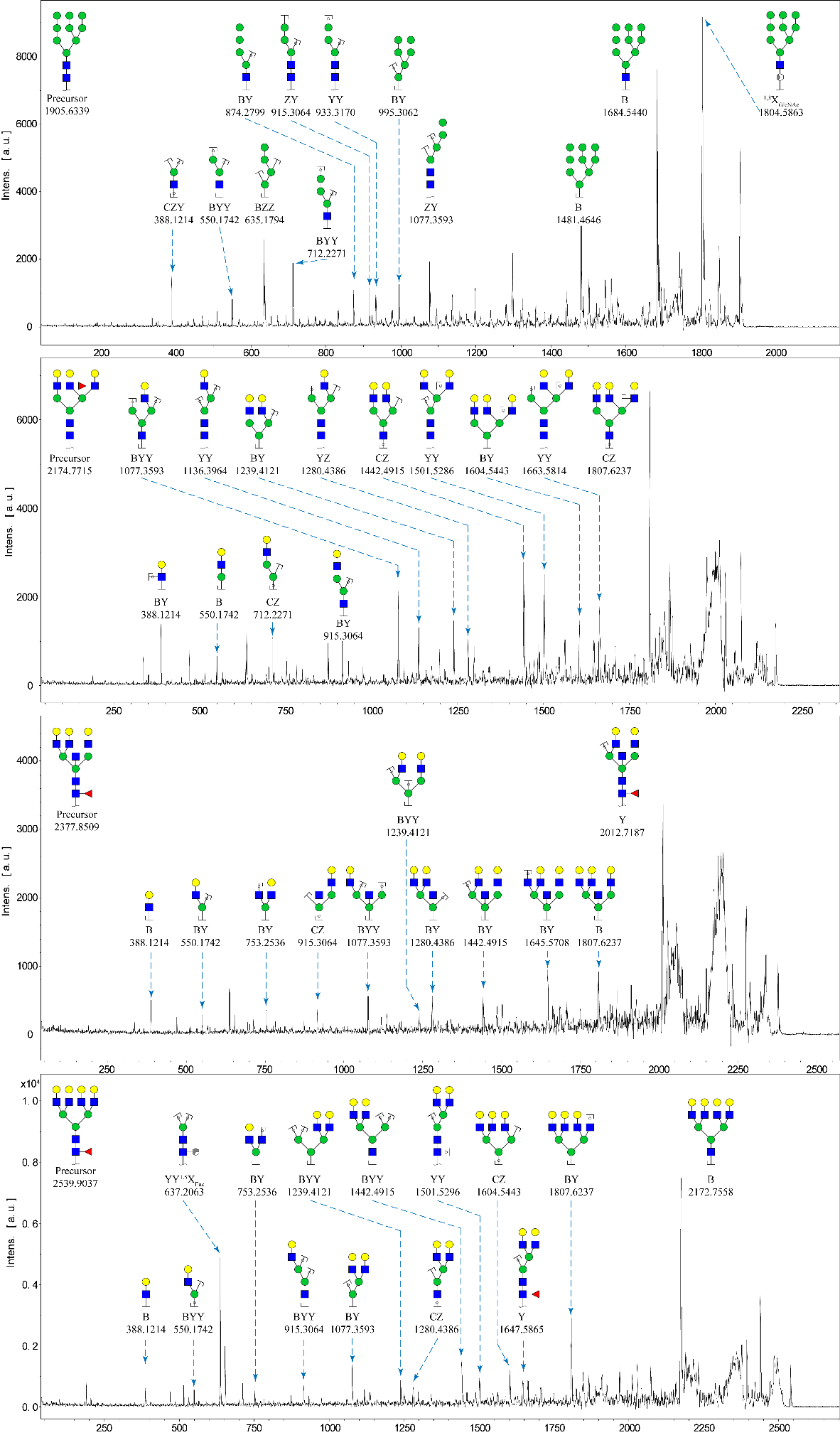


Fig. S1: MALDI-TOF/TOF-MS/MS analysis of N-glycan precursor ions in MS spectra.

Table S1: Proposed structures and their molecular ions of N-glycans from MCF7 cells under normoxia and hypoxia.

| **No.** | **Calculated m/z** | **Experimental**  **m/z** | **Glycan structures** | **MCF7** | |
| --- | --- | --- | --- | --- | --- |
|  |  |  |  | **Normoxia** | **Hypoxia** |
| 1 | 1257.423 | 1257.404 | 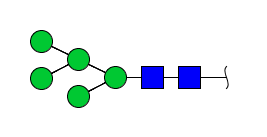 | 4.352±4.753 | 1.446±0.111 |
| 2 | 1419.458 | 1419.546 | 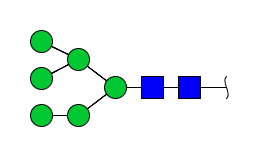 | 14.007±2.477 | 10.480±4.023 |
| 3 | 1565.533 | 1565.562 | 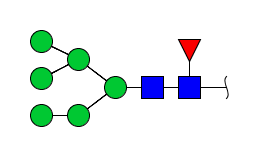 | 0.873±0.027 | 1.214±0.135 |
| 4 | 1581.528 | 1581.595 | 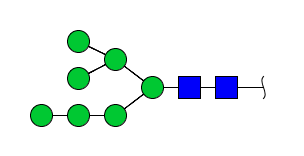 | 11.175±1.584 | 7.361±0.854 |
| 5 | 1727.586 | 1727.549 | 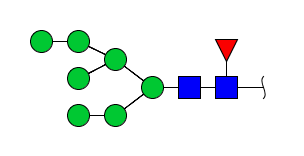 | ND | 0.915±0.077 |
| 6 | 1743.581 | 1743.626 | 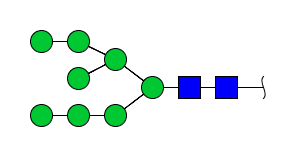 | 31.872±2.307 | 30.439±0.922 |
| 7 | 1809.639 | 1809.598 | 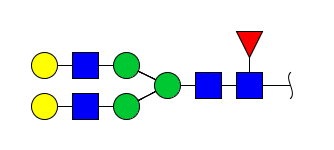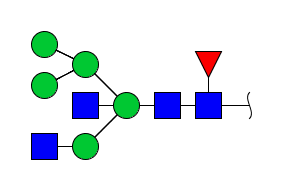 | 0.970±0.190 | ND |
| 8 | 1905.634 | 1905.801 | 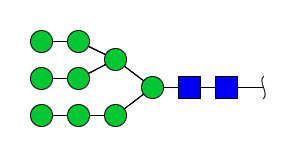 | 21.477±5.365 | 26.372±3.062 |
| 9 | 1955.697 | 1955.610 | 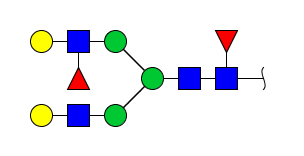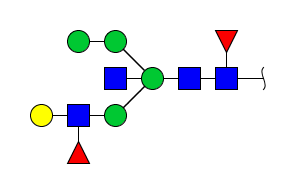 | 1.075±0.122 | 0.922±0.021 |
| 10 | 2067.687 | 2067.681 | 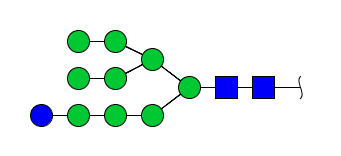 | 0.847±0.001 | 1.287±0.085 |
| 11 | 2101.755 | 2101.775 | 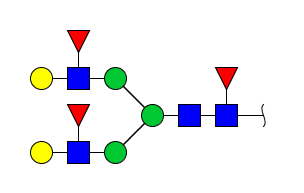 | 1.551±0.588 | 1.586±0.189 |
| 12 | 2174.772 | 2174.782 | 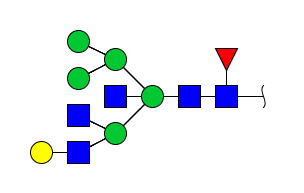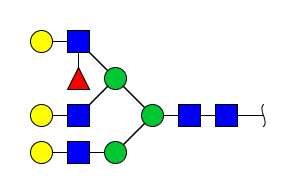  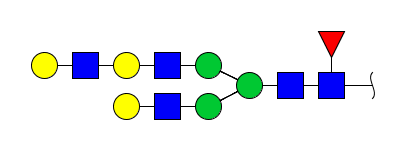 | 0.759±0.105 | ND |
| 13 | 2247.813 | 2247.886 | 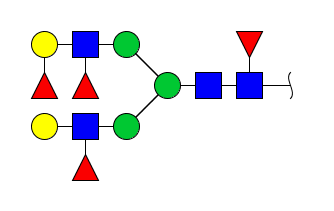 | 0.913±0.233 | 1.720±0.344 |
| 14 | 2320.829 | 2320.934 | 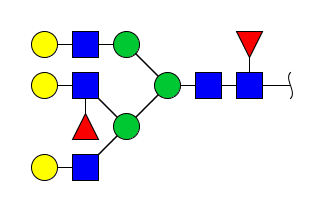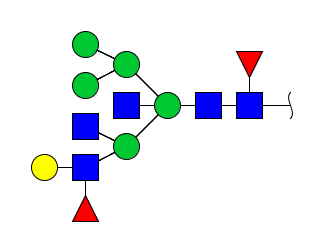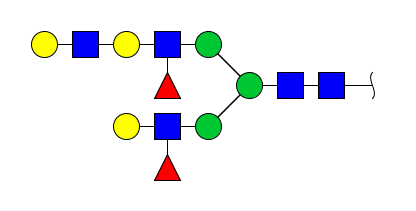 | 0.851±0.198 | 0.741±0.175 |
| 15 | 2377.851 | 2378.072 | 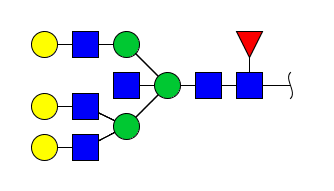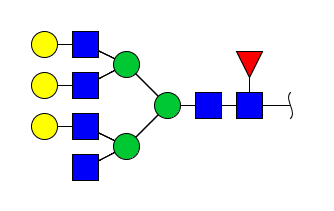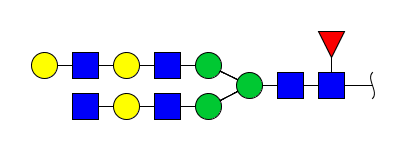 | 0.245±0.013 | ND |
| 16 | 2393.846 | 2393.947 | 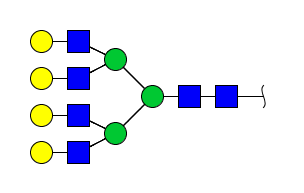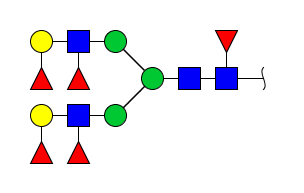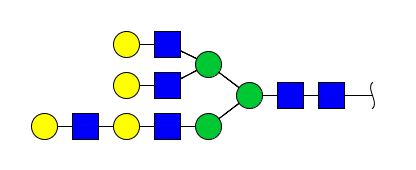 | 0.458±0.049 | 0.725±0.189 |
| 17 | 2466.887 | 2466.792 | 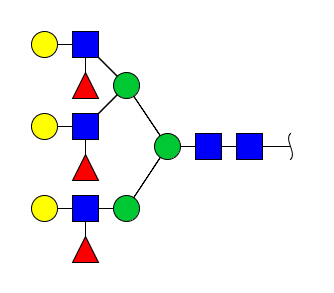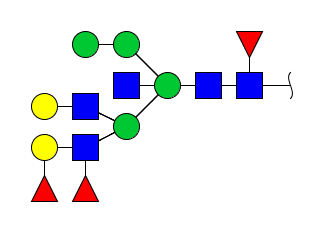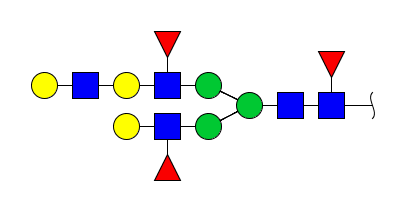 | 0.950±0.157 | 1.186±0.402 |
| 18 | 2523.909 | 2524.281 | 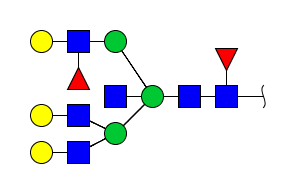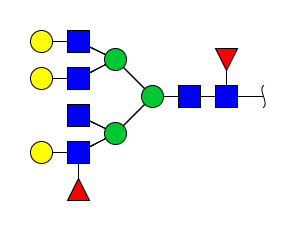 | 0.198±0.001 | ND |
| 19 | 2539.904 | 2539.978 | 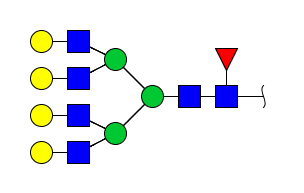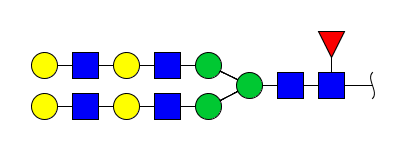 | 1.286±0.385 | 1.271±0.725 |
| 20 | 2612.945 | 2612.857 | 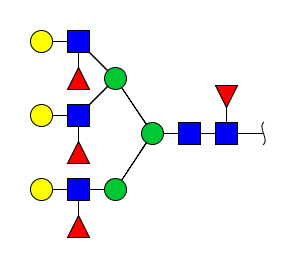 | 0.891±0.013 | 1.843±0.664 |
| 21 | 2669.967 | 2670.362 | 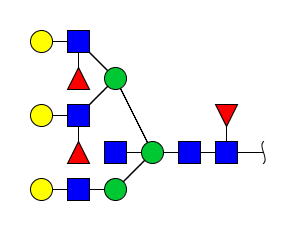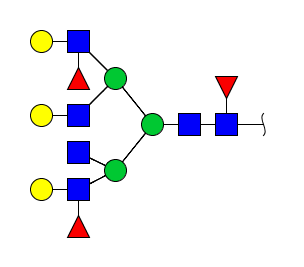 | 0.174±0.001 | ND |
| 22 | 2685.962 | 2686.060 | 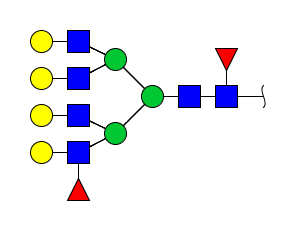 | 1.157±0.363 | 1.357±0.798 |
| 23 | 2758.978 | 2758.891 | 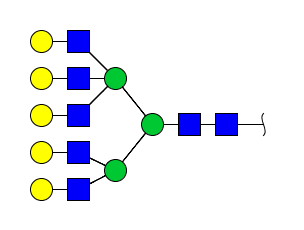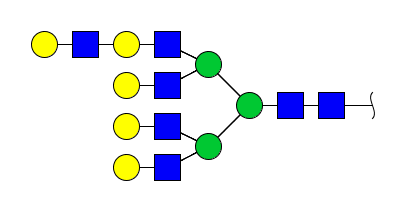 | 0.605±0.021 | 1.815±0.880 |
| 24 | 2832.02 | 2832.135 | 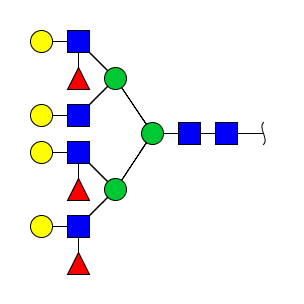 | 1.065±0.241 | 1.645±1.024 |
| 25 | 2905.036 | 2905.133 | 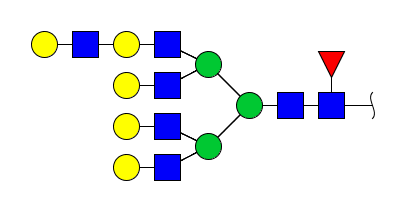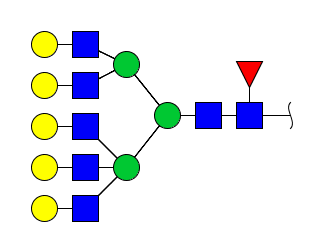 | 0.428±0.035 | 1.310±0.793 |
| 26 | 2978.077 | 2977.979 | 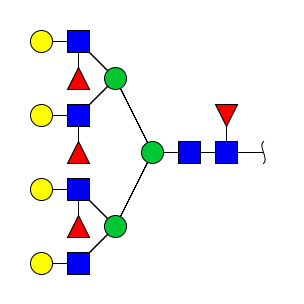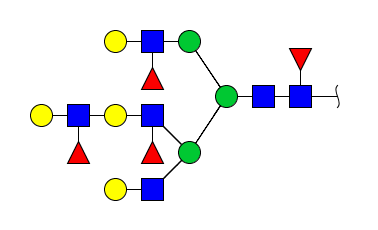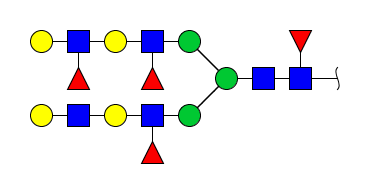 | 0.713±0.078 | 1.708±1.125 |
| 27 | 3051.119 | 3051.327 | 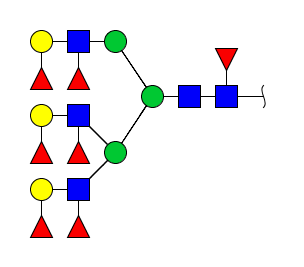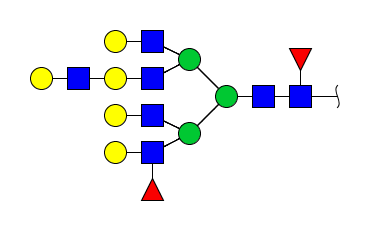 | 0.249±0.065 | 0.621±0.450 |
| 28 | 3124.135 | 3124.009 | 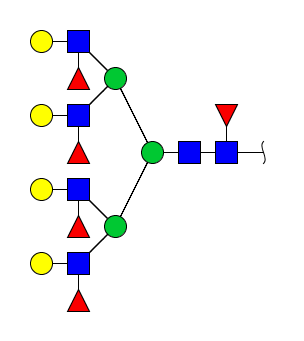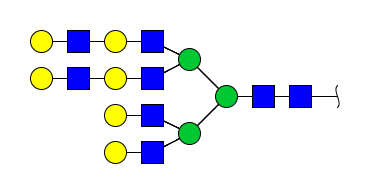 | 0.468±0.065 | 1.702±1.237 |
| 29 | 3197.152 | 3197.35 | 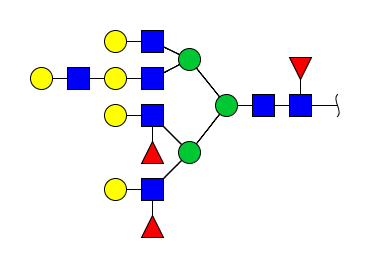 | 0.167±0.055 | ND |
| 30 | 3270.193 | 3270.419 | 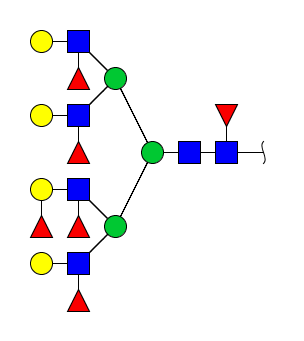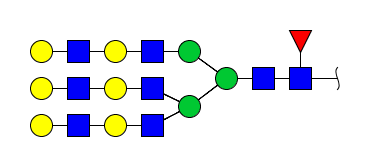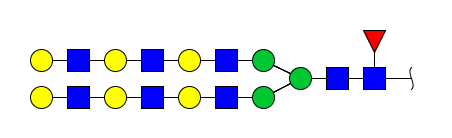 | 0.267±0.055 | 1.188±0.937 |
| 31 | 3343.21 | 3343.357 | 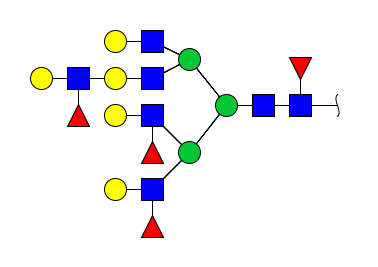 | 0.136±0.017 | ND |
| 32 | 3489.242 | 3489.433 | 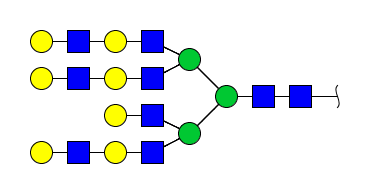 | 0.101±0.001 | ND |
| 33 | 3562.284 | 3562.471 | 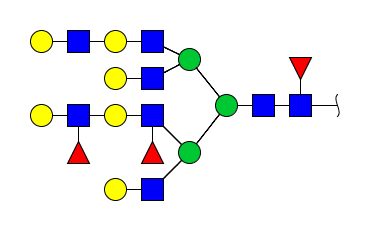 | 0.079±0.003 | 0.370±0.292 |
| 34 | 3635.3 | 3635.442 | 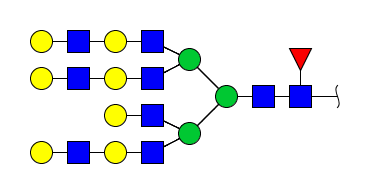 | 0.072±0.020 | ND |

Table S2: Proposed structures and their molecular ions of N-glycans from MDA-MB-231 cells under normoxia and hypoxia.

| **No.** | **Calculated m/z** | **Experimental**  **m/z** | **Glycan structures** | **MDA-MB-231** | |
| --- | --- | --- | --- | --- | --- |
|  |  |  |  | **Normoxia** | **Hypoxia** |
| 1· | 1079.375 | 1080.684 | 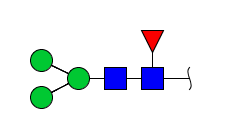 | ND | 0.514±0.026 |
| 2 | 1241.438 | 1241.713 | 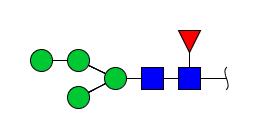 | 1.058±0.351 | ND |
| 3 | 1257.423 | 1257.537 | 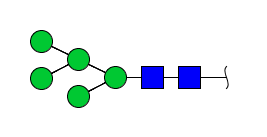 | 1.350±0.352 | 0.884±0.213 |
| 4 | 1403.481 | 1403.470 | 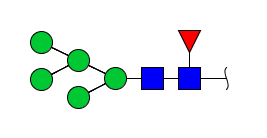 | 1.934±0.058 | 0.687±0.155 |
| 5 | 1419.458 | 1419.458 | 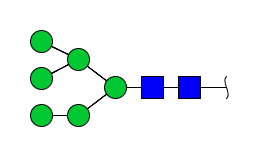 | 11.909±1.708 | 7.899±1.036 |
| 6 | 1565.533 | 1565.270 | 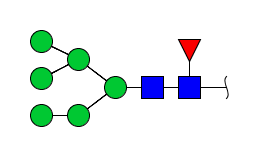 | 3.541±0.256 | 2.027±0.207 |
| 7 | 1581.528 | 1581.494 | 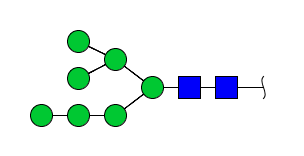 | 13.100±0.093 | 8.619±0.513 |
| 8 | 1727.586 | 1727.570 | 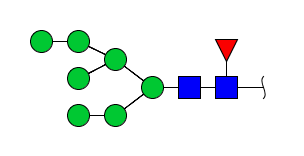 | 3.137±0.567 | 2.552±0.324 |
| 9 | 1743.581 | 1743.534 | 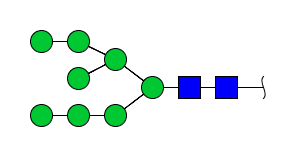 | 25.703±0.938 | 26.336±1.126 |
| 10 | 1757.592 | 1758.600 | 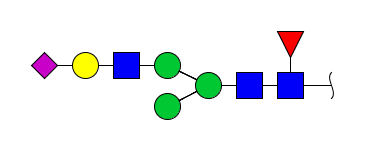 | 2.438±0.049 | ND |
| 11 | 1809.639 | 1809.648 | 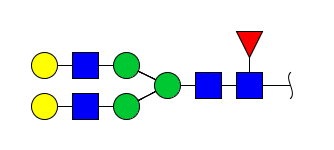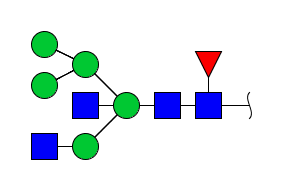 | 3.381±1.356 | 1.456±0.337 |
| 12 | 1891.692 | 1891.648 | 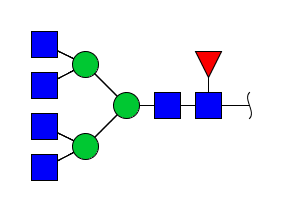  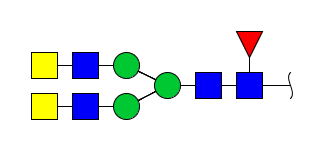  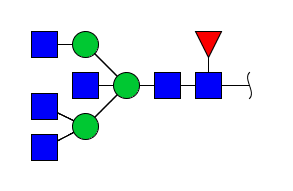 | ND | 0.689±0.116 |
| 13 | 1905.634 | 1905.573 | 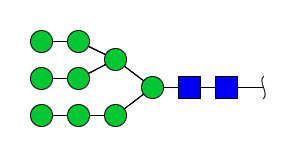 | 26.321±0.490 | 42.678±1.239 |
| 14 | 1919.645 | 1920.639 | 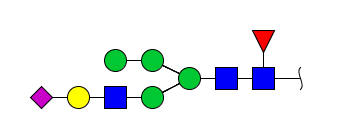 | 2.086±0.147 | ND |
| 15 | 1955.697 | 1955.691 | 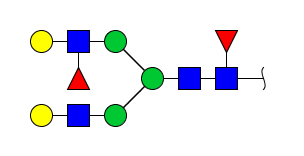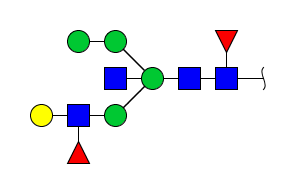 | 0.820±0.087 | 0.718±0.217 |
| 16 | 2067.687 | 2067.652 | 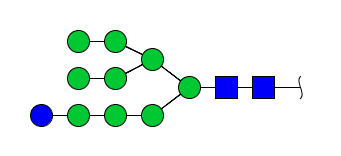 | 1.928±0.743 | 3.078±0.140 |
| 17 | 2101.755 | 2101.771 | 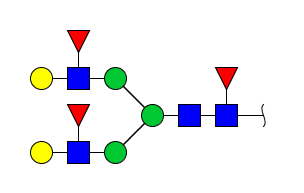 | 0.848±0.046 | 0.725±0.094 |
| 18 | 2174.772 | 2174.782 | 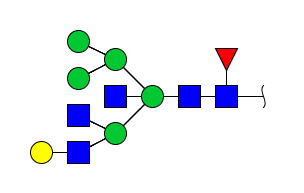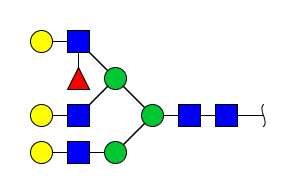  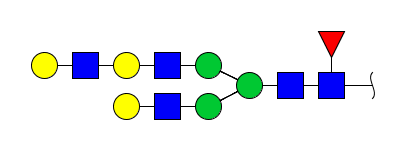 | 1.444±0.221 | 0.756±0.058 |
| 19 | 2539.904 | 2539.909 | 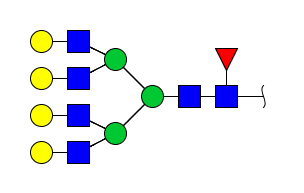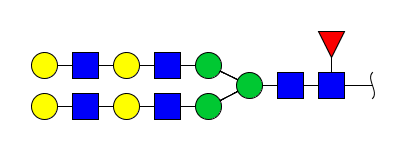 | 0.793±0.332 | 0.551±0.253 |

Table. S3: Glycan-binding specificities of the lectins used in lectin microarrays.

| **No.** | **Lectin** | **Binding structure** | **Fold change (Hypoxia/Normoxia)** | |
| --- | --- | --- | --- | --- |
|  |  |  | **MCF7** | **MDA-MB-231** |
| 1 | Jacalin | Galβ1-3GalNAcα-Ser/Thr(TF)  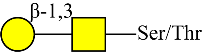  GalNAcα-Ser/Thr(T)  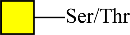 | 1.09 | 2.11 |
| 2 | ECA | Galβ-1,4GlcNAc  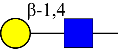 | 1.36 | 0.56 |
| 3 | HHL | Non-substituted α-1,6 Man  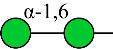 | 54.63 | 251.86 |
| 4 | WFA | GalNAcα/β1-3/6Gal  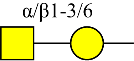 | 1.18 | 0.02 |
| 5 | GSL-II | GlcNAc  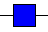  Galactosylated N-glycans  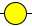 | -/- | 0.00 |
| 6 | MAL-II | Siaα2-3Galβ1-4Glc(NAc)  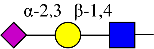 | ∞ | 0.34 |
| 7 | PHA-E | Bisecting GlcNAc  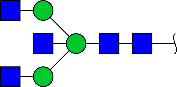 | 0.60 | 0.60 |
| 8 | PTL-I | αGalNAc and Gal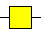, 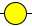 | ∞ | 0.60 |
| 9 | SJA | Terminal GalNAc and Gal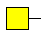,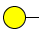 | 0.92 | 175.95 |
| 10 | PNA | Galβ1-3GalNAcα-Ser/Thr(T) 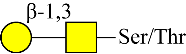 | 0.60 | 0.00 |
| 11 | EEL | Galα1-3(Fucα1-2)Gal  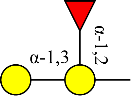 | 0.95 | ∞ |
| 12 | AAL | Terminal Fucα-1,6GlcNAc  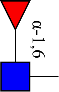  Fucα-1,3Galβ-1,4GlcNAc | 0.72 | 0.32 |
| 13 | LTL | Le^x^  Le^y^  Fucα-1,3GlcNAc (core) ； | 1.61 | 0.68 |
| 14 | MPL | αGalNAc | 6.65 | 0.96 |
| 15 | LEL | Poly-LacNAc    (GlcNAc)_n_ | 1.47 | 0.86 |
| 16 | GSL-I | αGalNAc, αGal  GalNAcα-Ser/Thr(Tn) | 0.86 | 156.70 |
| 17 | DBA | GalNAcα-Ser/Thr(Tn)    GalNAcα1-3Gal | 1.04 | 0.74 |
| 18 | LCA | Fucα-1,6GlcNAc (core) | 0.94 | 0.48 |
| 19 | RCA120 | Gal, GalNAc | 1.13 | 0.76 |
| 20 | STL | (GlcNAc)_n_ | 0.94 | 128.47 |
| 21 | BS-I | α-Gal, α-GalNAc | 0.68 | 0.06 |
| 22 | ConA | Branched and terminal Man,  terminal GlcNAc | 1.61 | 0.27 |
| 23 | PTL-II | Gal | 0.96 | 1.46 |
| 24 | DSA | GlcNAc | 1.09 | 2.33 |
| 25 | SBA | Terminal GalNAc (especially GalNAcα1-3Gal) | 1.74 | 0.00 |
| 26 | VVA | GalNAc  GalNAcα-Ser/Thr(Tn) | 0.84 | 1.87 |
| 27 | NPA | Non-substituted α-1,6Man | 0.95 | 1.76 |
| 28 | PSA | Fucα-N-acetylchitobiose-Man | 0.46 | 0.09 |
| 29 | ACA | Galβ1-3GalNAcα-Ser/Thr | 1.05 | 1.03 |
| 30 | WGA | Multivalent Sia  (GlcNAc)_n_ | 0.69 | 1.36 |
| 31 | UEA-I | Fucα1-2Galβ1-4Glc(NAc) | 0.88 | 1.38 |
| 32 | PWM | GlcNAc | 0.87 | 1.19 |
| 33 | MAL-I | Galβ-1,4GlcNAc | 0.45 | 0.78 |
| 34 | GNA | Terminalα-1,3 Man | 0.85 | 1.05 |
| 35 | BPL | Galβ1-3GalNAc | 1.04 | -/- |
| 36 | PHA-E+L | Bisecting GlcNAc    β1-6-GlcNAc branched N-glycan | 23.53 | -/- |
| 37 | SNA | Sia2-6Galβ1-4GlcNAc | 1.41 | 0.99 |
